# National Prevalence of Salmonella enterica Serotype Kentucky ST198 with High-Level Resistance to Ciprofloxacin and Extended-Spectrum Cephalosporins in China, 2013 to 2017

**DOI:** 10.1128/mSystems.00935-20

**Published:** 2021-01-12

**Authors:** Honghu Chen, Jingjie Song, Xianying Zeng, Dandan Chen, Rongchang Chen, Chen Qiu, Kai Zhou

**Affiliations:** a Shenzhen Institute of Respiratory Diseases, The First Affiliated Hospital (Shenzhen People’s Hospital), Southern University of Science and Technology, Shenzhen, China; b The Second Clinical Medical College of Jinan University (Shenzhen People’s Hospital), Jinan University, Shenzhen, China; c Zhejiang Provincial Center for Disease Control and Prevention, Hangzhou, China; d Guangxi Provincial Center for Disease Control and Prevention, Nanning, China; University of Illinois at Chicago

**Keywords:** *Salmonella enterica* serotype Kentucky, ST198, fluoroquinolone resistance, extended-spectrum cephalosporin resistance, *Salmonella* genomic island

## Abstract

Salmonella enterica serotype Kentucky is frequently associated with high-level fluoroquinolone resistance and has gained epidemiological importance globally. A retrospective screening was performed to understand the national prevalence of ciprofloxacin-resistant *S*. Kentucky in China. S. enterica strains (*n *=* *15,405) were collected within the frame of two national surveillance networks between 2013 and 2017. Thirty-three *S.* Kentucky strains were detected in 5 of 10 provinces, and 27 were assigned to sequence type 198 (ST198). The 27 isolates were multidrug resistant, with high-level resistance to ciprofloxacin, and 21 isolates were further resistant to extended-spectrum cephalosporins (ESCs). Phylogenomic analysis classified ST198 isolates into two clades (198.1 and 198.2), and recent occurrences of inter-/intraregion and interhost transmission were identified. Phylogenetic reconstruction with a global collection showed that one subclade of clade 198.2 was clustered with historical strains from Egypt, and the other one was clustered with strains from Southeast Asia. Isolates of clade 198.1 were clustered with strains isolated from North America. The various patterns of mutations detected in quinolone resistance-determining regions of GyrA and ParC are accordant with the phylogenetic structure. These findings indicate that our isolates may have various origins. SGI1 was exclusively detected in isolates of clade 198.2 with a highly mosaic structure, which were mainly identified as SGI1-K derivatives. Plasmid-mediated quinolone resistance genes *qnrS1* and *aac(6′)-Ib-cr* were identified in three isolates, and *bla*_CTX-M-9_ and *bla*_CTX-M-27_ were detected in 20 of 21 ESC-resistant isolates. This is the first report of the genetic and epidemiological characterization for the *S*. Kentucky epidemic clone ST198 in China, warranting the necessity of surveillance for the high-risk clone.

**IMPORTANCE** Ciprofloxacin and extended-spectrum cephalosporins are the choice for treatment of severe nontyphoidal S. enterica infections in adults. S. enterica serotype Kentucky ST198 has gained epidemiological importance globally, because the clone is frequently resistant to both of these high-level-resistance drug groups. The genetic and epidemiological characterization of *S.* Kentucky has been well studied in Western countries; however, the information is unclear for China. To fill in the gap, we here did a retrospective screening on a large collection in China, and ST198 isolates were systematically analyzed by whole-genome sequencing. Our study revealed that multidrug-resistant ST198 has spread in five provinces, and the occurrences of interregion and cross-host clonal disseminations were detected. Of note, phylogenomic analysis suggests that the Chinese isolates may have emerged with diverse origins, including Egypt, Southeast Asia, and North America. This study warrants the necessity of surveillance for the high-risk clone to prevent its further dissemination in China.

## Abstract (precis)

Ciprofloxacin and extended-spectrum cephalosporins are the choice for treatment of severe nontyphoidal S. enterica infections in adults. S. enterica serotype Kentucky ST198 has gained epidemiological importance globally, because the clone is frequently resistant to both of these high-level-resistance drug groups. The genetic and epidemiological characterization of *S.* Kentucky has been well studied in Western countries; however, the information is unclear for China.

## INTRODUCTION

Nontyphoidal Salmonella enterica (NTS) is among the most prevalent zoonotic pathogens, causing an estimated 93.8 million infections per year and resulting in 155,000 deaths globally (1). Numerous serotypes of NTS have been identified, and most of them inhabit animal intestines. However, a few commonly infect humans, including Salmonella enterica serotypes Enteritidis, Typhimurium, Virchow, Hadar, Heidelberg, Agona, and Indiana. Currently, the most common serotypes associated with human disease are *S.* Enteritidis and *S.* Typhimurium, while others appear to be becoming more visible on the global NTS landscape (2). Ciprofloxacin and extended-spectrum cephalosporins (ESCs) are the most frequent choices for the treatment of severe NTS infections in adults; thus, the emergence of resistance to ciprofloxacin and ESCs in S. enterica has become a serious public health concern. In 2017, fluoroquinolone-resistant *Salmonella* spp. and extended-spectrum-β-lactamase (ESBL)-producing *Enterobacteriaceae* were listed by the World Health Organization among the highest-priority pathogens posing a risk to human health.

Recently, the convergence of these high-level resistances has been found in a single serotype of S. enterica, Kentucky, which largely limits clinical treatment strategies (3). Epidemiological studies by using multilocus sequence typing (MLST) reveal that ciprofloxacin-resistant (CIP^r^) *S.* Kentucky is a single clone belonging to sequence type 198 (ST198). *S*. Kentucky ST198 has gained epidemiological importance globally (4–6), and the clone is frequently resistant to a variety of antibiotics, especially to ciprofloxacin at a high level (i.e., MIC > 4 mg/liter), a rare trait in NTS. CIP^r^ ST198 was first isolated in a French person who traveled back from Egypt in 2002 (5). Since then, this clone has been detected with high prevalence in Africa, the Middle East, and southern Asia, and it is widely disseminated to Europe and North America via travel-related infections (4–8). In addition, *S.* Kentucky is a foodborne pathogen, and domestic poultry has largely contributed to its global spread (most recently in South Asia and Europe) (3). *S.* Kentucky ST198 has been established in poultry in France, Poland, the United States, and Vietnam (3, 7–11). In some cases, the clone is also detected in bovines (10, 11). Therefore, CIP^r^ ST198 represents a significant risk to food safety and public health.

To understand the origin and evolutionary trajectory, a recent study analyzed a global collection of multidrug-resistant (MDR) *S*. Kentucky isolates by using whole-genome sequencing (WGS) for the first time (3). The results show that all of MDR *S*. Kentucky isolates belonged to ST198, which is estimated to have emerged circa 1989 in Egypt following the acquisition of the antimicrobial resistance (AMR)-associated *Salmonella* genomic island 1 (SGI1), conferring resistance to ampicillin, streptomycin, gentamicin, sulfamethoxazole, and tetracycline (3). In the early 2000s, amino acid substitutions in the quinolone resistance-determining regions (QRDRs) of GyrA and ParC conferring high-level resistance to fluoroquinolones were accumulated in ST198, further facilitating its wide dissemination (5). However, we noted that the WGS study lacked Chinese isolates, and only one historical genome retrieved from GenBank was included. More recently, a Chinese group performed an epidemiological investigation of *S*. Kentucky strains collected from clinical cases and the poultry supply chain between 2010 and 2016 in China (12). Using XbaI pulsed-field gel electrophoresis and MLST, the study determined that 35.0% (63/180) of *S*. Kentucky strains were ST198 and that 60.3% of ST198 isolates (38/63) were resistant to ciprofloxacin. However, due to the lack of WGS data, the study was unable to fully provide a genetic and epidemiological characterization of the isolates.

To fill in the knowledge gap, we analyzed in this study a national collection of *S*. Kentucky strains isolated from humans and food in China by using WGS. We aimed to provide a genetic and epidemiological characterization of *S.* Kentucky ST198 in China.

## RESULTS AND DISCUSSION

### *S.* Kentucky ST198 was detected in food and clinical samples in multiple provinces.

**FIG 1:**
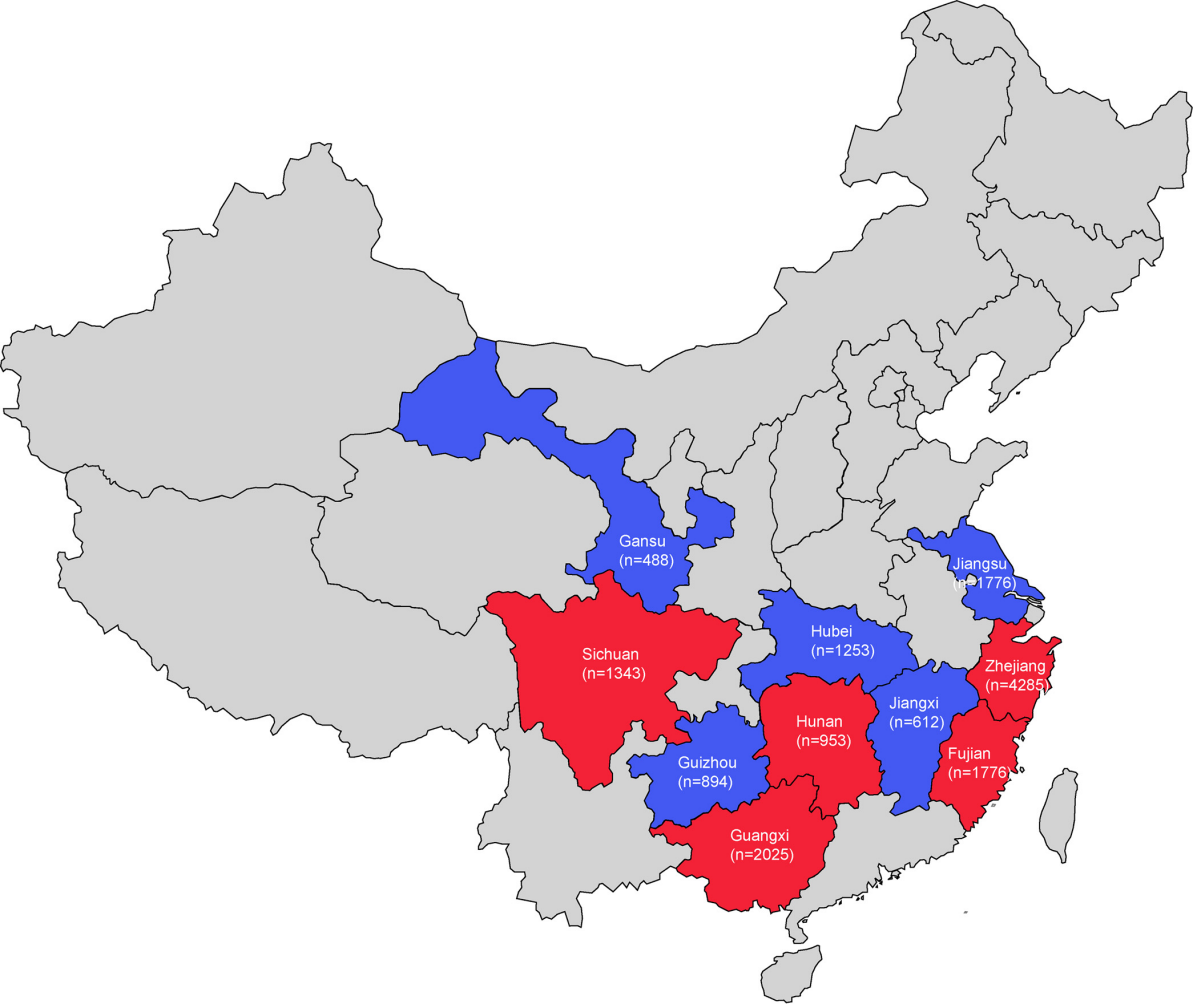
National prevalence of CIP^r^ *S.* Kentucky in China. The 10 provinces included in the surveillance are highlighted on the map, and the provinces where CIP^R^ *S.* Kentucky was identified are indicated in red. The number of isolates obtained from each province is shown.

A total of 12 *S.* Kentucky strains (0.097%) were detected by retrospectively screening 12,379 S. enterica strains isolated from patients with acute infectious diarrhea in 5 of 10 provinces (Fujian, Guangxi, Hunan, Sichuan, and Zhejiang) (Fig. 1). Eight of them were assigned to ST198, and the other four were ST314. Epidemiological investigations showed that none of the ST198-positive patients had records of traveling abroad within 3 months prior to their admissions, suggesting that acquisitions of *S*. Kentucky ST198 by these patients were not related to traveling abroad. This is different from the situation in Europe and North America, where more than half of ST198 infections are related to travel outside Europe and North America (5, 6).

As food has been identified as a potential major vehicle for infections by CIP^r^ ST198 in Europe, Africa, and North America, we further screened 3,026 serotyped S. enterica strains isolated from poultry, meat products, aquatic products, and eggs. Twenty-one *S*. Kentucky strains (0.69%) were isolated from poultry (*n *=* *17; chicken and duck) and meat products (*n *=* *4; pork, mutton, and pig liver), and they were collected from three provinces (Guangxi, Hunan, and Zhejiang), where positive human isolates were detected as well (Fig. 1). Nineteen of them were assigned to ST198, and the others were ST314. Together, our findings show that the positivity ratio for *S*. Kentucky ST198 among S. enterica isolates was 0.18% (27/15,405) in China, which is ca. 2-fold lower than that reported in a previous study (0.39% [40/16,247]) (12). This difference could be caused by different regions included in the sample collection. The data suggest that the positivity ratio for *S*. Kentucky ST198 was lower in China than in Europe (0.5% to 1%) and comparable to that in the United States (0.2%) (4, 5). The higher positivity ratio detected in food isolates than in clinical isolates is consistent with the previous findings that domestic poultry and poultry sources largely contribute to the global spread of ST198 (4, 12, 13). Additionally, our data showed that ST314 was the second most prevalent subpopulation in *S*. Kentucky.

### Occurrences of inter-/intraregion and cross-host transmission.

**FIG 2:**
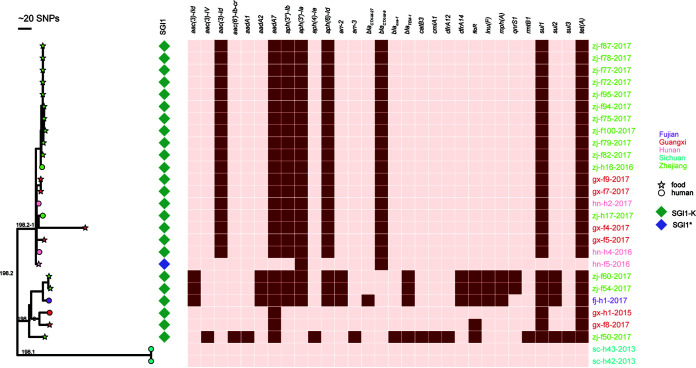
Core genome phylogeny of 27 *S.* Kentucky CIP^r^ ST198 strains. The mid-point-rooted phylogenetic tree was constructed by using the 4.3-Mb core genome. 198.1 and 198.2 are the two clades of ST198, and 198.2-1 and 198.2-2 are the two subclades of ST198.2. AMR genes and SGI1 detected are shown. The first two letters of the names of our isolates represent their origins, as follows: hn, Hunan; fj, Fujian; gx, Guangxi; sc, Sichuan; and zj, Zhejiang.

We performed a phylogenomic analysis on the 27 isolates to further understand their genetic relationship. The results based on 4.3-Mbp core genome showed the division of isolates into two clades: one clade consists of 25 isolates, and the other one includes the remaining 2 isolates (Fig. 2). A previous study showed that ST198 was diversified into two major clades, ST198.1 and ST198.2 (11). Clade 198.1 consists of isolates recovered from agricultural sources in the United States, and clade 198.2 is composed of human clinical isolates collected in Kuwait and the United States (11). In this study, the 25 isolates were classified into clade 198.2, and the other 2 isolates were assigned to clade 198.1 (Fig. 2), suggesting that clade 198.2 was much more prevalent than clade 198.1 in China. The two clades differed by 195 core genome single-nucleotide polymorphisms (SNPs), of which 147 were in coding regions. Clade 198.2 was further diversified into two subclades (198.2-1 and 198.2-2) differing by 44 SNPs, of which 40 were in coding regions.

A few intraregional clonal transmission events were detected, including (i) 10 strains (zj-f72, zj-f75, zj-f77, zj-f79, zj-f78, zj-f82, zj-f87, zj-f94, zj-f95, and zj-f100) isolated from chicken in Zhejiang province differing by 0 to 6 SNPs, (ii) 2 strains (zj-f54 and zj-f60) isolated from chicken in Zhejiang province differing by 4 SNPs, (iii) 2 strains (gx-f7 and gx-f9) isolated from pork and duck in Guangxi province with identical genetic backgrounds (i.e., 0 SNPs), and (iv) 2 strains (sc-h42 and sc-h43) isolated from human in Sichuan province with identical genetic backgrounds. Very few SNPs (≤10 SNPs) detected among strains in each event suggests a recent occurrence. Further epidemiological investigation showed that the chicken isolates were from the same slaughterhouse in Zhejiang province, the two strains (gx-f7 and gx-f9) isolated from pork and duck were obtained from two cities in Guangxi province, and the two human isolates (sc-h42 and sc-h43) were obtained from the same family. The results indicate that most intraregional clonal transmission events are caused by epidemiological contacts.

Interregional clonal transmission events were also identified: 10 chicken strains (zj-f72, zj-f75, zj-f77, zj-f79, zj-f78, zj-f82, zj-f87, zj-f94, zj-f95, and zj-f100) isolated in Zhejiang province shared a close relation with those isolated in Guangxi (gx-f7 and gx-f9; 17 to 19 SNPs) and Hunan (hn-f5; 14 to 18 SNPs) provinces. Of note, the recent occurrences of cross-host transmissions were detected among a few strains collected in the same region: a clinical strain (zj-h16) collected in Zhejiang province was highly similar to the 10 chicken isolates described above (5 to 7 SNPs), and 2 clinical strains (hn-h2 and hn-h4) collected in Hunan province shared a close relation with the chicken isolate hn-f5 (7 to 9 SNPs). Taken together, these data suggest that ST198 has clonally disseminated in China.

### Origins of Chinese ST198 isolates might be diverse.

**FIG 3:**
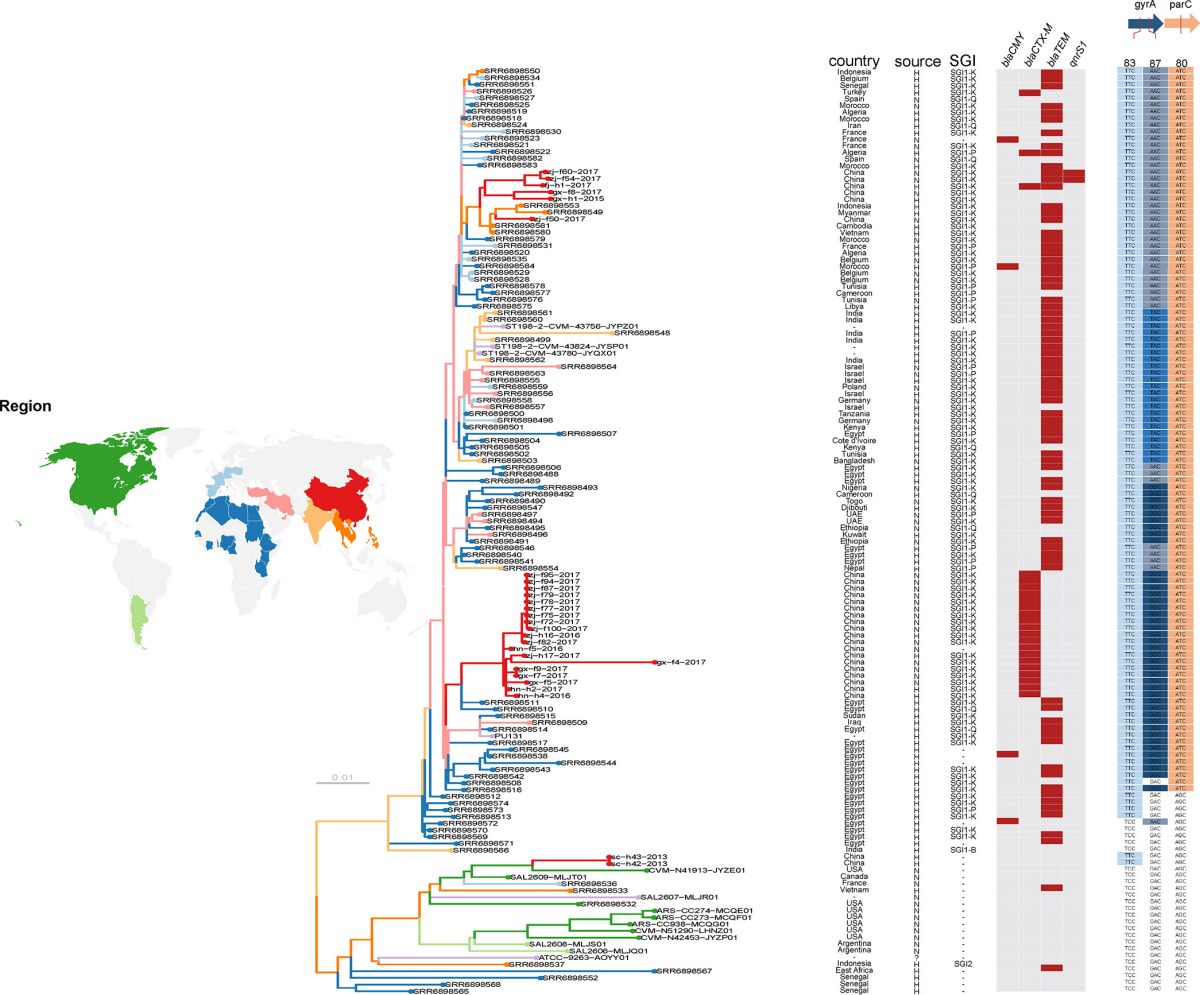
Core genome phylogeny of global *S.* Kentucky CIP^r^ ST198. The phylogenetic tree is mid-point rooted. A total of 116 genomes of ST198 representing a global data set were retrieved from GenBank and were included in the analysis in this study. Leaf nodes are colored by region of origin (see map). Colored branches indicate inferred geographical distribution of internal branches. Data columns indicate country of origin, source of isolate (H, human; N, nonhuman; ?, unknown), SGI type, obtained resistance genes of beta-lactams and quinolones, and quinolone resistance-related codons, with resistance-associated alleles highlighted. The first two letters of the name of our isolates represent their origins; the year of sampling is shown by the number behind the isolate name.

To further understand the genetic relationship of isolates collected from China and other countries, we performed phylogenetic analysis with additional 116 genomes retrieved from GenBank, representative of a global collection (3, 13). The 19 strains of subclade 198.2-1 clustered with historical strains isolated from Egypt (26 to 86 SNPs) (Fig. 3). The amino acid mutations in QRDRs detected in these 19 strains (*gyrA*, TCC to TTC, Ser83Phe; *gyrA*, GAC to GGC, Asp87Gly; and *parC*, AGC to ATC, Ser80Ile) were identical to those in the Egypt historical strains (Fig. 3). The six strains of subclade 198.2-2 clustered with strains isolated from Southeast Asia (Cambodia, Indonesia, Myanmar, and Vietnam) (30 to 52 SNPs) (Fig. 3). They shared identical amino acid mutations in the QRDRs (*gyrA*, TCC to TTC, Ser83Phe; *gyrA*, GAC to AAC, Asp87Asn; and *parC*, AGC to ATC, Ser80Ile), of which the mutation of *gyrA* codon 87 was different from that in subclade 198.2-1. The two isolates of clade 198.1 clustered with strains isolated from North America (Canada and the United States) (65 to 72 SNPs). Mutation of the QRDR (*gyrA*, TCC to TTC, Ser83Phe) was exclusively identified in two Chinese isolates from clade 198.1 (Fig. 3).

It is suggested that high-level resistance of ST198 strains to fluoroquinolones likely first emerged in Egypt in the early 2000s; this resistance is mainly caused by a combination of three amino acid substitutions in the QRDRs of *gyrA* and *parC* (5). GyrA-Ser83Phe (TCC to TTC) is the first mutation obtained by ST198, circa 1992, followed by ParC-Ser80Ile (AGC to ATC) circa 1996. Ciprofloxacin resistance appeared after the occurrence of mutations GyrA-Asp87Gly (GAC to GGC), Asp87Asn (GAC to AAC or), and Asp87Tyr (GAC to TAC) (3, 5). Previous epidemiological studies demonstrated that strains carrying the GyrA-Asp87Tyr (TAC) mutation mainly spread from Egypt and Northern Africa into East Africa, Middle Africa, South Asia, Europe, and Western Asia, and those with the GyrA-Asp87Asn (AAC) mutation spread to Southeast Asia, Europe, and West Africa (3–5). Taken together, the data suggest that the Chinese isolates collected in this study might have various geographic origins and evolved locally.

### Various resistomes were detected in ST198 isolates.

All ST198 isolates (*n *=* *27) collected in this study were MDR (Table 1). They showed high-level resistance to ciprofloxacin (MIC ≥ 8 mg/liter) and nalidixic acid (MIC > 128 mg/liter) and remained susceptible to carbapenems, colistin, and tigecycline. Twenty-one isolates were further resistant to ESCs (MIC ≥ 16 mg/liter). Analysis of resistance genes revealed that the resistomes of 18 isolates of subclade 198.2-1 were identical, including *aaCA5* [*aac(3)-Id*], *aadA7*, *aph(3″)-Ia*, *strA* [*aph(3′)-Ib*], *strB* [*aph (6’)-Id*], *bla*_CTX-M-9_, *sul1*, and *tet(A)*, while only two resistance genes [*bla*_CTX-M-9_ and *aph(3′’)-Ia*] were detected in the other isolate (hn-f5) from 198.2-1. The consistent resistome detected in 198.2-1 isolates further demonstrates that a recent clonal dissemination occurred among three provinces (Zhejiang, Guangxi, and Hunan). The ESBL gene *bla*_CTX-M-9_ carried by the 19 isolates is located on the chromosome. More resistance genes were detected in isolates of 198.2-2, except for gx-h1 and gx-f8, than in isolates of 198.2-1, and no acquired resistance genes were found in the two isolates of 198.1 (Fig. 2). Of concern, plasmid-mediated quinolone resistance (PMQR) genes *aac(6′)-Ib-cr* and *qnrS1* were detected in zj-f50, zj-f60, and zj-f54. The *qnrS1* gene was flanked by a Tn*3*-like transposon, a relic of an IS*2*-like insertion sequence upstream, and a truncated *tnpR* gene downstream. The genetic context of *qnrS1* is identical to that carried by plasmid pINF5 (GenBank accession number AM234722) detected in an *S.* Infantis strain (see Fig. S1 in the supplemental material). However, a conjugation assay failed in getting transconjugants with ciprofloxacin resistance. It is supposed that recombination via Tn*3* might mediate the capture of *qnrS1* by the plasmid (14). Previous studies show that PMQR genes are rarely carried by ST198 isolates, while they seem more common in Chinese isolates. A recent report revealed that *aac(6′)-Ib-cr* (19/63) and *qnr*-type genes (9/63) are the prevalent PMQR genes among ST198 isolates collected in China (12). The emergence of PMQR genes would further largely facilitate the wide dissemination of quinolone resistance among ST198 clones.

**TABLE 1:** Antibiotic resistance profile of *S.* Kentucky ST198 isolates collected in this study^*a*^

Sample ID	Source	Yr	Location	KZ	CXM	CTX	CAZ	FOX	AZM	FEP	IPM	MEM	COL	SMX	TMP	CIP	TET	AZI	NAL	CHL	TGC	AMP	GEN
zj-f54	Chicken	2017	Zhejiang	S	I	S	S	R	S	S	S	S	S	R	R	R	R	R	R	R	I	R	R
zj-f60	Chicken	2017	Zhejiang	S	I	S	S	I	S	S	S	S	S	R	R	R	R	R	R	R	I	R	R
zj-f72	Chicken	2017	Zhejiang	R	R	R	S	S	R	R	S	S	S	R	S	R	R	S	R	S	S	R	R
zj-f75	Chicken	2017	Zhejiang	R	R	R	S	S	I	R	S	S	S	R	S	R	R	S	R	S	S	R	R
zj-f77	Chicken	2017	Zhejiang	R	R	R	S	S	I	R	S	S	S	R	S	R	R	S	R	S	S	R	R
zj-f78	Chicken	2017	Zhejiang	R	R	R	I	S	I	R	S	S	S	R	S	R	R	S	R	S	S	R	R
zj-f79	Chicken	2017	Zhejiang	R	R	R	S	S	I	R	S	S	S	R	S	R	R	S	R	S	S	R	R
zj-f82	Chicken	2017	Zhejiang	R	R	R	S	S	I	R	S	S	S	R	S	R	R	S	R	S	S	R	I
zj-f87	Chicken	2017	Zhejiang	R	R	R	S	S	I	I	S	S	S	R	S	R	R	S	R	S	S	R	R
zj-f94	Chicken	2017	Zhejiang	R	R	R	S	S	R	R	S	S	S	R	S	R	R	S	R	S	S	R	I
zj-f95	Chicken	2017	Zhejiang	R	R	R	S	S	I	R	S	S	S	R	S	R	R	S	R	S	I	R	I
zj-f100	Chicken	2017	Zhejiang	R	R	R	S	S	I	R	S	S	S	R	S	R	R	S	R	S	S	R	R
zj-h16	Human	2016	Zhejiang	R	R	R	S	S	I	R	S	S	S	R	S	R	R	S	R	S	S	R	R
zj-h17	Human	2017	Zhejiang	R	R	R	I	S	I	R	S	S	S	R	S	R	R	S	R	S	S	R	R
hn-f5	Chicken	2016	Hunan	R	R	R	S	S	I	R	S	S	S	S	S	R	S	S	R	S	S	R	S
hn-h2	Human	2017	Hunan	R	R	R	S	I	R	R	S	S	S	R	S	R	R	S	R	S	S	R	R
hn-h4	Human	2016	Hunan	R	R	R	S	S	I	R	S	S	S	R	R	R	R	R	R	R	S	R	R
fj-h1	Human	2017	Fujian	R	R	R	R	I	R	R	S	S	S	R	R	R	R	R	R	R	I	R	R
sc-h42	Human	2013	Sichuan	S	I	S	S	S	S	S	S	S	S	R	R	R	R	R	R	S	S	R	R
sc-h43	Human	2013	Sichuan	S	S	S	S	S	S	S	S	S	S	R	S	R	S	S	R	S	S	R	S
gx-h1	Human	2015	Guangxi	S	S	S	S	S	S	S	S	S	S	R	S	R	R	S	R	S	S	S	S
gx-f4	Mutton	2017	Guangxi	R	R	R	R	S	R	R	S	I	S	R	S	R	R	S	R	S	S	R	R
gx-f5	Pork	2017	Guangxi	R	R	R	S	S	S	R	S	S	S	R	S	R	R	S	R	S	S	R	R
gx-f7	Pork	2017	Guangxi	I	R	R	S	S	I	I	S	S	S	R	S	R	R	S	R	S	S	R	R
gx-f8	Pig liver	2017	Guangxi	S	I	S	S	S	S	S	S	S	S	R	S	R	R	S	R	R	S	S	S
gx-f9	Duck	2017	Guangxi	R	R	R	S	S	S	R	S	S	S	R	S	R	R	S	R	S	S	R	R

a Abbreviations: ID, identifier; KZ, cefazolin; CXM, cefuroxime; CTX, cefotaxime; CAZ, ceftazidime; FOX, cefoxitin; AZM, aztreonam; FEP, cefepime; IPM, imipenem; MEM, meropenem; COL, colistin; SMX, sulfamethoxazole; TMP, trimethoprim; CIP, ciprofloxacin; TET, tetracycline; AZI, azithromycin; NAL, nalidixic acid; CHL, chloramphenicol; TGC, tigecycline; AMP, ampicillin; GEN, gentamicin; S, susceptible; I, intermediate; R, resistant. Breakpoints (in milligrams per liter) are as follows: KZ, S ≤ 1, I = 2, and R ≥ 4; CXM, S ≤ 8, I = 16, and R ≥ 32; CTX, S ≤ 1, I = 2, and R ≥ 4; CAZ, S ≤ 4, I = 8, and R ≥ 16; FOX, S ≤ 8, I = 16, and R ≥ 32; AZM, S ≤ 4, I = 8, and R ≥ 16; FEP, S ≤ 2, I = 4 to 8, and R ≥ 16; IPM, S ≤ 1, I = 2, and R ≥ 4; MEM, S ≤ 1, I = 2, and R ≥ 4; COL, S ≤ 2, I = 4, and R ≥ 8; SMX, S ≤ 256 and R ≥ 512; TMP, S ≤ 8 and R ≥ 16; CIP, S ≤ 1, I = 2, and R ≥ 4; TET, S ≤ 4, I = 8, and R ≥ 16; AZI, S ≤ 16 and R ≥ 32; NAL, S ≤ 16 and R ≥ 32; CHL, S ≤ 8, I = 16, and R ≥ 32; TGC, S ≤ 2, I = 4, and R ≥ 8; AMP, S ≤ 8, I = 16, and R ≥ 32; and GEN, S ≤ 4, I = 8, and R ≥ 16.

### The mosaic structure of SGI1 detected in ST198 isolates.

**FIG 4:**
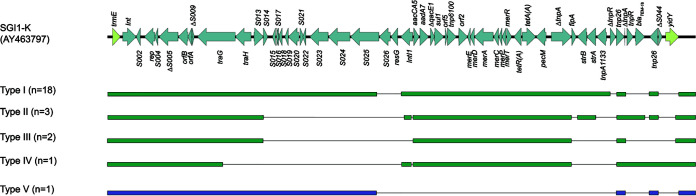
SGI variations in *S*. Kentucky ST198 isolates collect in this study. The prototype sequence of SGI-K (GenBank accession number AY463797) is shown as a reference. Five variations were detected in our collection, and the number of each variation is shown in parentheses. Black lines indicate the deletion regions corresponding to the reference. Type I to IV variations are supposed to be SGI-K derivatives (in green); type V is not an SGI-K derivative (in purple), since the mercuric ion resistance module was not detected. SGI and chromosome genes (*trmE* and *yidY*) are indicated by different colors.

A novel SGI1-K variant, carrying a mercuric ion resistance module, was detected in the 18 isolates of 198.2-1. Compared with the prototype of SGI1-K, few deletions were detected in the variant, including *S026-resG*, *tnpR* of Tn*5393*, a *bla*_TEM-1b_-bearing Tn*2*, and *ΔS044* (Fig. 4). The resistance region was completely deleted in the SGI1 carried by the other isolate of 198.2-1 (hn-f5), including the features of SGI1-K, e.g., gene cassettes *aacCA5* to *aadA7* and the *mer* module (15). This indicates the occurrence of rapid intraclone evolution. Another SGI1-K variant was identified in three isolates of 198.2-2 (zj-f54, zj-f60, and fj-h1), in which 14 backbone genes (*S015* to ∼*S026*, *resG*, and *ΔS044*), *tnpR* of Tn*5393*, and IS*1133* were deleted (Fig. 4). More deletions were found in the SGI1-K variant carried by three strains, including Tn*5393* and Tn*2* in gx-h1 and gx-f8 and *S011*, *S012*, and Tn*5393* in zj-f50. SGI1 was not detected in the two isolates of 198.1. Of note, among SGI-1-positive isolates, zj-f50 was the only one that maintained *ΔS044* and *yidY* (Fig. 4). This was different from the global isolates reported previously (3), suggesting that Chinese isolates might undergo selection and evolve locally. It has been reported that the structure of SGI1 is highly mosaic in *S.* Kentucky ST198, mainly caused by IS*26* mediating the gain or loss of genes in the island, resulting in numerous variants (15). We suppose that the flexible structure of SGI1 detected in *S. Kentucky* ST198 may confer an adaptive advantage to this high-risk clone.

### Plasmidome of ST198 isolates.

Analysis of the plasmidome revealed that 23 strains carried at least one plasmid, covering six different known replicon types (ColRNAI, Col156, Col440I, IncHI2/2A, IncI1, and IncQ1). ColRNAI-type plasmid was the most prevalent one (18/27), followed by Col156 (5/27) and Col440I (4/27). Three different plasmid incompatibility types (IncHI2/2A, IncI1, and IncQ1) were identified in three isolates. We identified that the IncHI2/2A-type plasmid carried by zj-f50 carried numerous AMR genes, including *aac(3)-IV*, *aph(4)-I*, *sul2*, *sul3*, *aadA1*, *cmlA1*, *rmtB1*, *floR*, *aac(6′)-Ib-cr*, *bla*_OXA-1_, *catB3*, *arr-3*, and *dfrA12*. It has been known that IncHI2-type plasmids are frequently involved in the acquisition of AMR genes (16–18). Of note, IncHI2/2A-type plasmids frequently mediate the dissemination of AMR genes across the most prevalent S. enterica serotypes (including Kentucky) (19). This thus raises the concern that the high-risk clone ST198 may become more resistant resulting from the dissemination of such MDR IncHI2/2A-type plasmids in the near future, at least in China.

### Conclusion.

We here first report the genomic characterization of the *S.* Kentucky epidemic clone CIP^r^ ST198 with ESC resistance in China. The prevalence ratio of CIP^r^ ST198 is relatively low in human and food isolates; however, the occurrence of interregion and interhost clonal disseminations highlights the necessity of surveillance for the high-risk clone to prevent its further dissemination in China. Of particular concern, the emergence of plasmid-borne AMR genes in ST198 would further worsen the clinical treatment.

## MATERIALS AND METHODS

### Bacterial isolates collected in this study.

A total of 12,379 serotyped S. enterica strains isolated from patients with acute infectious diarrhea were collected by 10 provincial Center for Disease Control and Prevention (CDC) sites during a national surveillance for foodborne pathogens between 2013 and 2017 (Fig. 1), and 3,026 serotyped S. enterica strains isolated from food were collected by these CDC sites during a national surveillance for food contaminants and their virulence factors in the same area and period (Fig. 1). Epidemiological data were recorded for each isolate.

### Antibiotic susceptibility testing (AST).

The broth dilution method was performed for all *S.* Kentucky (8:i:z6) strains to determine the MIC values of cefazolin (0.25 to 32 mg/liter), cefuroxime (0.5 to 64 mg/liter), cefotaxime (0.5 to 64 mg/liter), ceftazidime (0.5 to 64 mg/liter), cefoxitin (0.5 to 64 mg/liter), aztreonam (0.25 to 32 mg/liter), cefepime (0.25 to 32 mg/liter), imipenem (0.125 to 16 mg/liter), meropenem (0.125 to 16 mg/liter), colistin (0.25 to 16 mg/liter), trimethoprim-sulfamethoxazole (0.125/2.375 to 16/304 mg/liter), trimethoprim (8 to 1,024 mg/liter), ciprofloxacin (0.03 to 4 mg/liter), tetracycline (1 to 64 mg/liter), azithromycin (0.5 to 64 mg/liter), nalidixic acid (1 to 128 mg/liter), chloramphenicol (1 to 128 mg/liter), tigecycline (0.25 to 32 mg/liter), ampicillin (0.5 to 64 mg/liter), and gentamicin (0.25 to 32 mg/liter). The results were interpreted using the Clinical and Laboratory Standards Institute (CLSI) document M100 breakpoints (20). Strains were defined as high-level ciprofloxacin resistant if the MIC was >4 mg/liter and as high-level resistant to ESCs (i.e., cefotaxime or ceftazidime) if the MIC was ≥16 mg/liter. All isolates were further tested for ESBL production. Isolates showing a ≥3 2-fold concentration decrease in an MIC for either cefotaxime (0.5 to 64 mg/liter) or ceftazidime (0.5 to 64 mg/liter) tested in combination with clavulanic acid (4 mg/liter) versus the MIC of the agent when tested alone were considered ESBL producing.

### Compilation of genomic data set.

A global data set of *S*. Kentucky ST198 genomes was retrieved from the National Center for Biotechnology Information submitted from two previous studies (3, 13), which are the only genomic data available currently. In total, 116 ST198 genomes were able to be obtained from GenBank and were included in the analysis in this study.

### WGS and data analysis.

Genomic DNA was extracted using a Gentra Puregene Yeast/Bact kit (Qiagen, San Francisco, CA). Paired-end libraries (2 × 125 bp) were constructed and sequenced on a HiSeq 2500 instrument (Illumina, San Diego, CA). *De novo* assembly was performed after quality trimming (quality score ≥ 20) by using CLC Genomics Workbench v10.0 (Qiagen, Hilden, Germany). AMR genes were identified in the assembled genomes using ABRicate v0.9.8 (https://github.com/tseemann/abricate) to query the ResFinder database v3.2 (21) with thresholds of 90% identity and 60% coverage. Plasmid replicon typing was performed using PlasmidFinder (22) with 95% identity and 60% coverage. MLST was analyzed by using the MLST tools (https://cge.cbs.dtu.dk/services/MLST/). Detection of SGI sequences was mainly dependent on BLASTn. The contigs of each genome were BLAST searched against the reference of SGI-1K (GenBank accession number AY463797). If the alignment coverage and nucleotide similarity were ≥90%, a reference-like SGI was supposed to exist. The alignment with a coverage range of 60 to 90% was checked manually to determine the existence of backbone genes of SGI. The genetic context of antimicrobial resistance genes was identified by BLAST searching the contig sequence harboring target genes in GenBank, and the synteny analysis was performed using Easyfig (http://mjsull.github.io/Easyfig/). Reconstruction of individual plasmid sequences from draft genome assemblies was performed using the tool MOB-recon and the clustered plasmid reference databases of MOB-suite (23).

Short reads of 27 *S.* Kentucky ST198 isolates were mapped to the reference genome PU131 (GenBank accession number CP026327), and 116 genome sequences retrieved from GenBank were aligned, using the pipeline Snippy (https://github.com/tseemann/snippy) to identify single-nucleotide polymorphisms (SNPs) with the “-mapqual 60 -basequal 13 -mincov 10 -minqual 100 -maxsoft 10” switches. SNPs were filtered to exclude those present in repeat regions and mobile genetic elements as previously described (3), and SNPs in recombinant regions were excluded by using Gubbins v1 (24) with default settings. The resulted high-quality SNPs were used for phylogenetic reconstructions by using RAXML v8.1.23 (25), with the model GTRGAMMA and 100 bootstrap replicates.

### Conjugation assay.

Conjugative transfer of plasmids was evaluated using plasmid-free rifampin-resistant E. coli strain EC600 as the recipient with various donor/recipient ratios (1:1, 4:1, and 1:4) at 37°C. Transconjugants were selected on MacConkey agar with rifampin (600 mg/liter) and ciprofloxacin (1 mg/liter and 4 mg/liter). Selected transconjugants were verified by matrix-assisted laser desorption ionization–time of flight mass spectrometry (MALDI-TOF MS) (Bruker Daltonik GmbH, Bremen, Germany) and amplification of the *qnrS1* gene.

### Accession number(s).

WGS data of 27 isolates have been deposited in GenBank under BioProject number PRJNA543407 (accession numbers VCKR00000000 to VCJT00000000).

10.1128/mSystems.00935-20.1 FIG S1 Genetic context of *qnrS1* identified in this study. The sequence of pINF5 (GenBank accession number AM234722) including *qnrS1* is as referenced here. Download FIG S1, JPG file, 2.8 MB. Copyright © 2021 Chen et al. 2021 Chen et al. This content is distributed under the terms of the Creative Commons Attribution 4.0 International license.
